# Risk factors for inappropriate blood requisition among hospitals in Tanzania

**DOI:** 10.1371/journal.pone.0196453

**Published:** 2018-05-17

**Authors:** Wilhellmuss I. Mauka, Tara B. Mtuy, Michael J. Mahande, Sia E. Msuya, Innocent B. Mboya, Abdul Juma, Rune N. Philemon

**Affiliations:** 1 Department of Epidemiology and Biostatistics, Institute of Public Health, Kilimanjaro Christian Medical University College, Moshi, Tanzania; 2 National Blood Transfusion Services, Dar es Salaam, Tanzania; German Red Cross Blood Donation Center, GERMANY

## Abstract

**Background:**

Blood is a critical aspect of treatment in life saving situations, increasing demand. Blood requisition practices greatly effect sufficient supply in blood banks. This study aimed to determine the risk factors for inappropriate blood requisition in Tanzania.

**Methods:**

This was a cross sectional study using secondary data of 14,460 patients’ blood requests from 42 transfusion hospitals. Primary data were obtained by using cluster-sampling design. Data were analysed using a two-level mixed-effects Poisson regression to determine fixed-effects of individual-level factors and hospital level factors associated with inappropriate blood requests. P-value <0.05 (2-tails) was considered statistically significant.

**Results:**

Inappropriate requisition was 28.8%. Factors significantly associated with inappropriate requisition were; reporting pulse rate and capillary refill decrease the risk (RR 0.74; 95% CI 0.64, 0.84) and (RR 0.73; 95% CI 0.63, 0.85) respectively and the following increased the risk; having surgery during hospital stay (RR 1.22; 95% CI 1.06, 1.4); being in general surgical ward (RR 3.3; 95% CI 2.7, 4.2), paediatric ward (RR 1.8; 95% CI 1.2, 2.7), obstetric ward (RR 2.5; 95% CI 2.0, 3.1), gynaecological ward (RR 2.1; 95% CI 1.5, 2.9), orthopaedics ward (RR 3.8; 95% CI 2.2, 6.7). Age of the patient, pallor and confirmation of pre-transfusion haemoglobin level were also significantly associated with inappropriate requisition. Majority of appropriate requisitions within the wards were marked in internal medicine (91.7%) and gynaecological wards (77.8%).

**Conclusions:**

The proportion of inappropriate blood requests was high. Blood requisition was determined by clinical and laboratory findings and the ward patients were admitted to. Adherence to transfusion guidelines is recommended to assure the best use of limited blood supply.

## Background

In Sub Saharan Africa (SSA), blood and blood components transfusion have become a common practice especially in contemporary medicine, aiding in life saving situations [1,2]. This has resulted in an increasing demand of safe blood and its products especially among children and mothers during and after delivery [3–7]. Due to an increase in demand, scarcity of blood in blood banks is eminent [8,9]

Ineffective blood transfusion services have been contributing to maternal mortality in sub-Saharan Africa [10] as 25% of maternal mortality is attributed to obstetric hemorrhage [11]. SSA is facing a weak blood donation infrastructure which is compounded with social-economic challenges [12–14]. In addition to these factors, blood requisition practices can exacerbate insufficient blood supply in blood banks [15–17], depending on adherence to transfusion guidelines. Furthermore the effect of over-ordering of blood, results in increased and unnecessary patient costs including costs of blood and testing prior to transfusion (e.g. grouping and cross-matching) [18,19].

WHO guidelines on clinical use of blood and blood products has been adopted by several countries including Tanzania [20–22]. The guidelines are evidence based so that those who are in need of blood should have access to blood transfusion. This study aimed to determine the proportion and risk factors associated with inappropriate blood requisition among hospitals in Tanzania in 2013.

## Methods

### Data source

The parent study was carried out in Tanzania from June 17^th^ through September 27^th^ 2013. The study population included all patients’ blood transfusion requests submitted at hospital blood banks during the study period. Sampling and data collection procedures are explained in detail elsewhere [23].

The study protocol was reviewed and approved by the Tanzania National Institute of Medical Research, the Zanzibar Medical Research and Ethics Committee (ZAMREC) and the Institutional Review Board at Centers for Disease Control and Prevention (CDC).

### Current study

#### Study design and population

This was a secondary data analysis of a hospital based cross sectional survey. The analysis included all patients’ blood transfusion requests for whole blood (WB) and packed red blood cells (PRBC), submitted to hospital blood banks during the study period. Requests for blood grouping without cross-matching test were excluded from analysis.

### Sample size and power

This study involved 14,460 blood requests from 42 transfusion hospitals that met the current study criteria, to estimate the overall proportion of inappropriate transfusion with 95% confidence with margin error of ±2%. The sample size had a power more than 99% to detect a 5% difference in the proportion of inappropriate requisition.

#### Study variables

**Fig 1:**
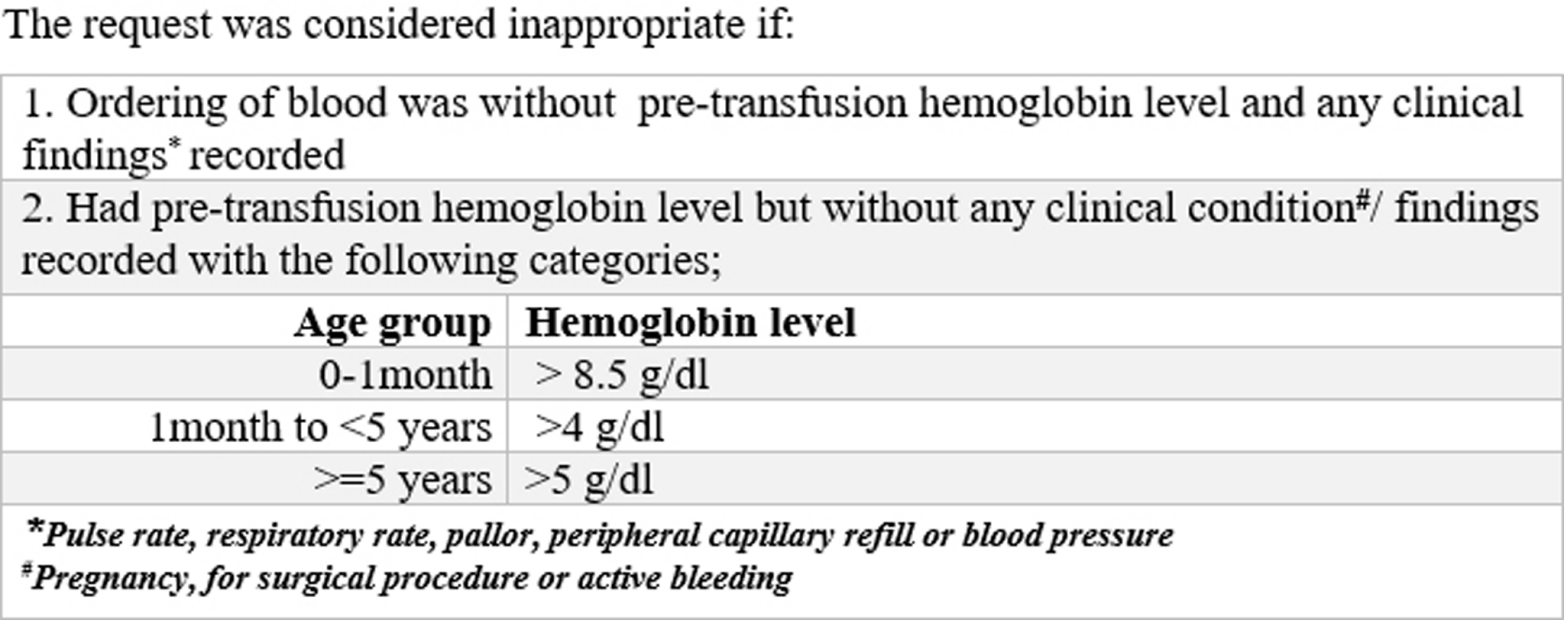
Criteria for assessment of inappropriate requisition of PRBC/Whole blood.

The dependent variable in this analysis was inappropriate blood request, a binary response (yes/no), determined through relevant criteria (Fig **1**) [24,25]. Independent variables were socio-demographic characteristics (age and sex of the patient and hospital area-urban/rural). Pre-admission history (admitted ward, diagnosis, underlying cause of anaemia), pre-transfusion laboratory test (pre-transfusion haemoglobin level-Hb) and vital signs (blood pressure, pulse rate, respiratory rate), patient pre-transfusion signs and symptoms (active bleeding, consciousness, cardiac failure, cold extremities, decreased capillary refill, respiratory distress, large liver or spleen, pallor and tachycardia), and transfusion information (number of units requested/ issued/ not, transfused/not, cross-matched).

#### Data processing method

**Fig 2:**
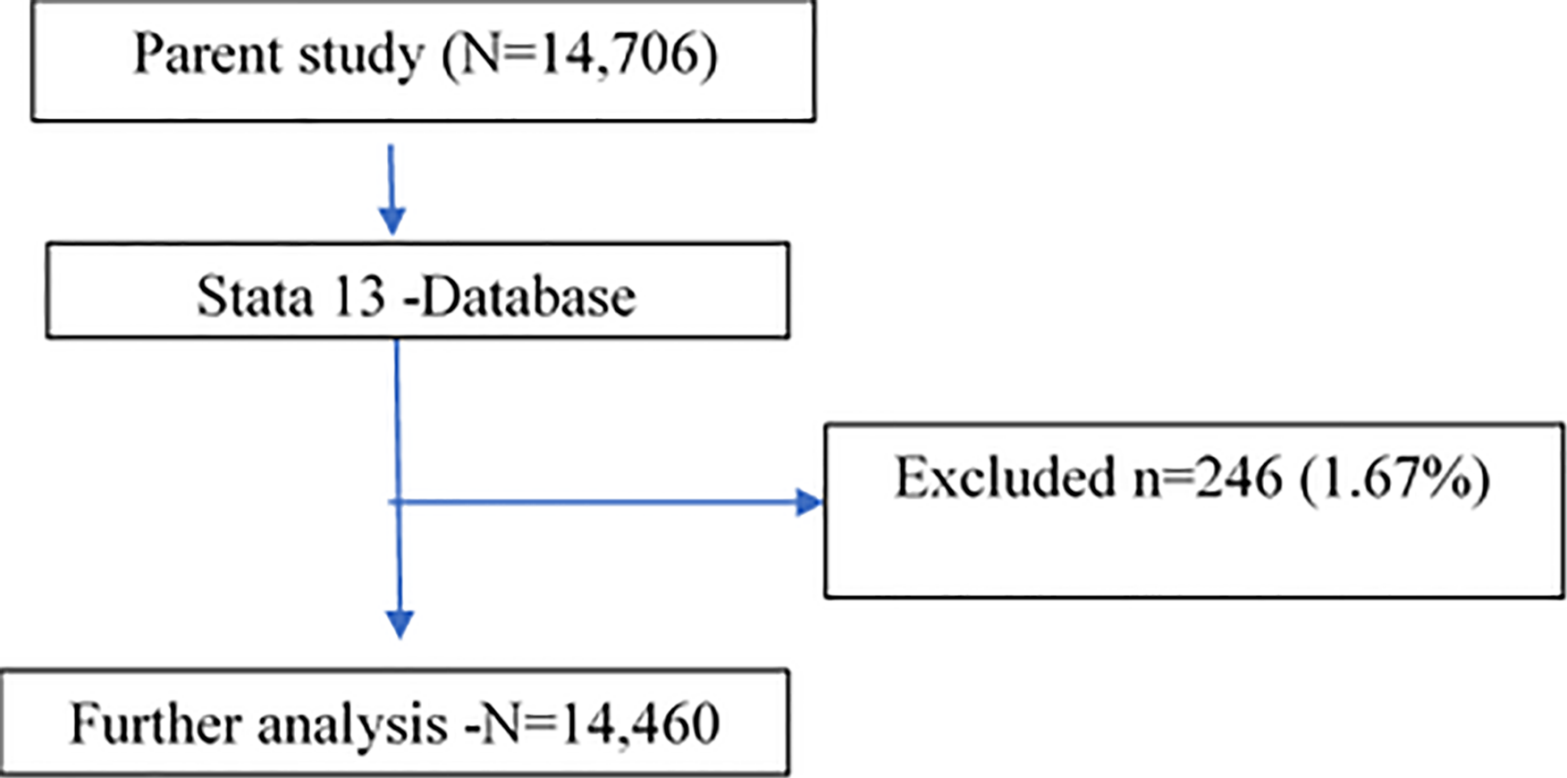
Data sampling process.

All data were extracted from survey database (Excel spreadsheet) into Stata version 13.1 Stata-Corp LP, for cleaning and further analysis (**Fig 2)**.

#### Data description

The data were hierarchical or multilevel structures such that blood requests were nested within patients and patients nested within hospitals, and hospitals nested within clusters. Thus requests of the same patient were more similar among each other than requests from different patients. Furthermore, individual patients within the same cluster (hospitals) could be more similar to each other than patients among all hospitals and variance of observations might not be constant across risk factors.

#### Data analysis

Data were summarized into frequency, median (IQR) and percentages. Clinical presentation of the patients and inappropriate blood requests were described considering clustering effect to get average distribution percentage between and within the hospitals. Bivariate analysis was done for testing association between main outcome (inappropriate requesting) and potential exposures (risk factors). Chi-square test of independence was used.

Multilevel mixed-effects generalized linear model was used to test the effect size of individual and hospital factors on inappropriate blood request and estimate the between-cluster variability of effect of inappropriate blood request [26–29].

#### Univariate multilevel analysis

The analysis involved all variables associated with inappropriate blood request in the bivariate analysis (p<0.05).

#### Multivariable multilevel analysis

The analysis involved entering all the variables with P-value <0.05 in univariate analysis. Backward a two-level mixed-effects Poison regression was done in order to determine the factors which significantly predict the risk of inappropriate blood request with probability of elimination at 0.05.

To test the significance of clusters (hospitals) on inappropriate requests, the empty model (a model with only outcome of interest but without any explanatory variables) was run with cluster and then an empty model without clusters.

Then a multilevel multivariable model was computed to account for the hierarchical structure of the data and clustering of responses at the different levels. Six models were run, whereby models 2–6 were compared against the empty model by deploying Akaike Information Criterion.

Intra-Class Correlation (ICC) was used to determine the proportion of the variance that is due to clusters. ICC was calculated using between-cluster variance and within-cluster variance (П^2^ /3). This was used to compare the successive models by looking at the decline of the ICC to explain the variability in risk of inappropriate request. The model was checked for possible confounders and interaction for covariates by using Likelihood Ratio test.

Complete case analysis was used as variables with the greatest missing data could still hold minimum sample size required, hence the study had sufficient power for identifying potential differences.

#### Ethical consideration

Ethical clearance was obtained from the Kilimanjaro Christian Medical College Research Ethical Committee and permission to use the data from the parent study was obtained from National Blood Transfusion Services. Confidentiality of participants’ information was assured using participant identification numbers.

## Results

### Baseline characteristics of patients

Blood requests from 11,189 patients were ordered from hospital blood banks. Among 10,544 patients whose ages were recorded, the median age was 25, ranging from 6 to 38 years. Among 9,713 recorded haemoglobin levels, the median haemoglobin level was 6g/dl ranging between 4.4 and 8.6g/dl. Of 11,153 patients, more than half 6,998 (62.8%), were females. One-third, 3,743 (33.5%), of patients were from Eastern zone of the country and 853 (7.6%) were from Zanzibar, which had the least number of patients with blood requests. Of 11,153 records, 3,323 (29.8%) patients were admitted in paediatric medical wards followed by adult medical wards 2,691 (24. 1%). From a total of 12,373 reported clinical signs, more than two thirds of patients, 7,924 (64.7%), had pallor, followed by 2,092 (16.9%) with active bleeding and the least reported clinical sign was decreased capillary refill, 54 (0.4%). Patient characteristics are as shown in Table 1.

**Table 1:** Baseline characteristics of patients (11,189).

Characteristics	Median (IQR) n (%)	
Median Age (IQR) years (n = 10,544)	25 (6–38)	
Median pulse rate (IQR) bpm (n = 7,035)	86 (80–102)	
Median Haemoglobin level (IQR) g/dl (n = 9,713)	6 (4.4–8.6)	
Age group (Years) (N = 10,544) *		
0–4	2,264	(21.5)
5–14	1,143	(10.8)
15–24	1,627	(15.4)
25–34	2,245	(21.3)
35–44	1,475	(14)
45–54	686	(6.5)
55–64	485	(4.6)
65+	619	(5.8)
Sex (n = 11,153)*		
Male	4,155	(37.3)
Female	6,998	(62.7)
**Hospital ownership**		
Government	8,555	(76.5)
Private	2,634	(23.5)
**Hospital area**		
Rural	3,044	(21.1)
Urban	11,416	(78.9)
**Number of patients by zone**		
Eastern-Zone	3,743	(33.5)
Lake Zone	1,566	(14)
Northern	1,083	(9.7)
Southern Highland	1,393	(12.5)
Southern	977	(8.7)
Western	1,574	(14.1)
Zanzibar	853	(7.6)
**Clinical signs (n = 12,997)** **		
Active bleeding	2,092	(16.9)
Abnormal thinking or unconscious	353	(2.9)
Features of Cardiac failure	283	(2.3)
Cold extremities	75	(0.6)
Decreased capillary refill	54	(0.4)
Respiratory Distress	684	(5.5)
Pallor	7,924	(64)
Tachycardia	908	(7.3)
Ward type (11,153) *		
Adult surgery	1,453	(13)
Adult medical	2,691	(24.1)
Paediatric Medical	3,323	(29.8)
Paediatric surgical	281	(2.5)
Intensive Care Unit	141	(1.3)
Obstetrics	2,263	(20.3)
Gynaecological	958	(8.6)
Orthopaedics	34	(0.3)

*There are missed data

**More than one clinical sign could be presented by on patient

### Distribution of characteristics of blood requests

Underlying causes of anaemia were reported in 9577 requests from 42 hospitals. Of these, 3,483 (37%) requests were due to malaria, 2,363 (24.7%) due to maternal haemorrhage and the least was tuberculosis 151 (1.6%). Among the 42 hospitals, in at least one of their blood requests, 41 (97.2%) had reported malaria and 40 (95.2%) reported maternal haemorrhage as the underlying cause of anaemia **(**Table **2****)**. Furthermore, within the hospitals which documented the underlying cause of anaemia in at least one of their blood request forms, on average 45.9% of their *requests* were due to malaria, 23.5% due to maternal haemorrhage. The rest of the underlying causes of anaemia are distributed as shown in Table **2**.

**Table 2:** Reported clinical presentation of patients’ blood requests (N = 14,460).

Variables	Overall		Hospitals		Average Within Hospitals
	n	(%)	N = 42	(%)	(%)
Underlying the cause of anaemia (n = 9577)*					
Cancer	987	(10.5)	34	(81)	(11.8)
HIV-related	1,033	(11.0)	33	(78.6)	(12)
Malaria	3,483	(37.0)	41	(97.6)	(45.9)
Maternal Haemorrhage	2,363	(24.7)	40	(95.2)	(23.5)
Non-trauma surgery	555	(5.9)	26	(61.9)	(5.5)
Sickle Cell Disease	475	(5.1)	35	(83.3)	(6.2)
Trauma	399	(4.2)	34	(81)	(4.5)
Tuberculosis	151	(1.6)	30	(71.4)	(2.4)
**Clinical Signs**					
Tachycardia (n = 14,170)*					
No	5,043	(35.6)	41	(97.6)	(35.1)
Yes	9,127	(64.2)	41	(97.6)	(67.3)
Tachypnea (n = 14,531)*					
No	5,164	(36.2)	38	(90.5)	(31.4)
Yes	9,101	(63.8)	41	(97.6)	(73.4)
Haemoglobin level (n = 8998) **					
Normal haemoglobin level	1,081	(20.6)	40	(95.2)	(18.1)
Anaemia	7,917	(79.4)	42	(100)	(82.8)
**Recorded Clinical Signs (N = 14,460)**					
**Cold Extremities**					
Yes	3,518	(24.3)	36	(85.7)	(23.7)
No	10,942	(75.7)	42	(100)	(79.7)
**Capillary refill**					
Yes	3,220	(22.3)	32	(76.2)	(22.9)
No	11,240	(77.7)	42	(100)	(82.8)
**Tachycardia**					
Yes	5,668	(39.9)	41	(97.6)	(32)
No	8,534	(60.1)	42	(100)	(68)
**Pallor**					
Yes	12,379	(85.6)	42	(100)	(81.1)
No	2,081	(14.4)	39	(95.2)	(19.8)
**Respiratory distress**					
Yes	6,331	(43.8)	41	(97.6)	(34.9)
No	8,129	(56.2)	41	(97.6)	(67.6)
Inappropriate blood requests (N = 12,204)*					
No	8,687	(71.2)	42	(100)	(71.2)
Yes	3,517	(28.8)	42	(100)	(28.8)

*There are missed data

**Categorization based on age and sex, hence number has decreased for those

Pulse rate was recorded in 14,170 requests from 42 hospitals. Overall 64.2% of recorded pulse rates showed signs of tachycardia whereby 97.6% of hospitals had patients with tachycardia in at least one of its blood requests. Within the hospitals, an average of 67.3% of requests which pulse rate recorded revealed signs of tachycardia. Among 8998 requests which recorded patients’ haemoglobin levels, 7,917 (79.4%) had anaemia while 1,081 (20.6%) had normal haemoglobin levels.

Different clinical signs were reported in 14,460 blood requests, with more than three quarters of blood requests (85.6%) reported pallor. All 42 (100%) hospitals, reported pallor in at least in one of their blood requests. Within hospitals, on average 81.1% reported pallor in at least one of its blood requests. Other clinical signs are shown in Table **2**.

**Fig 3:**
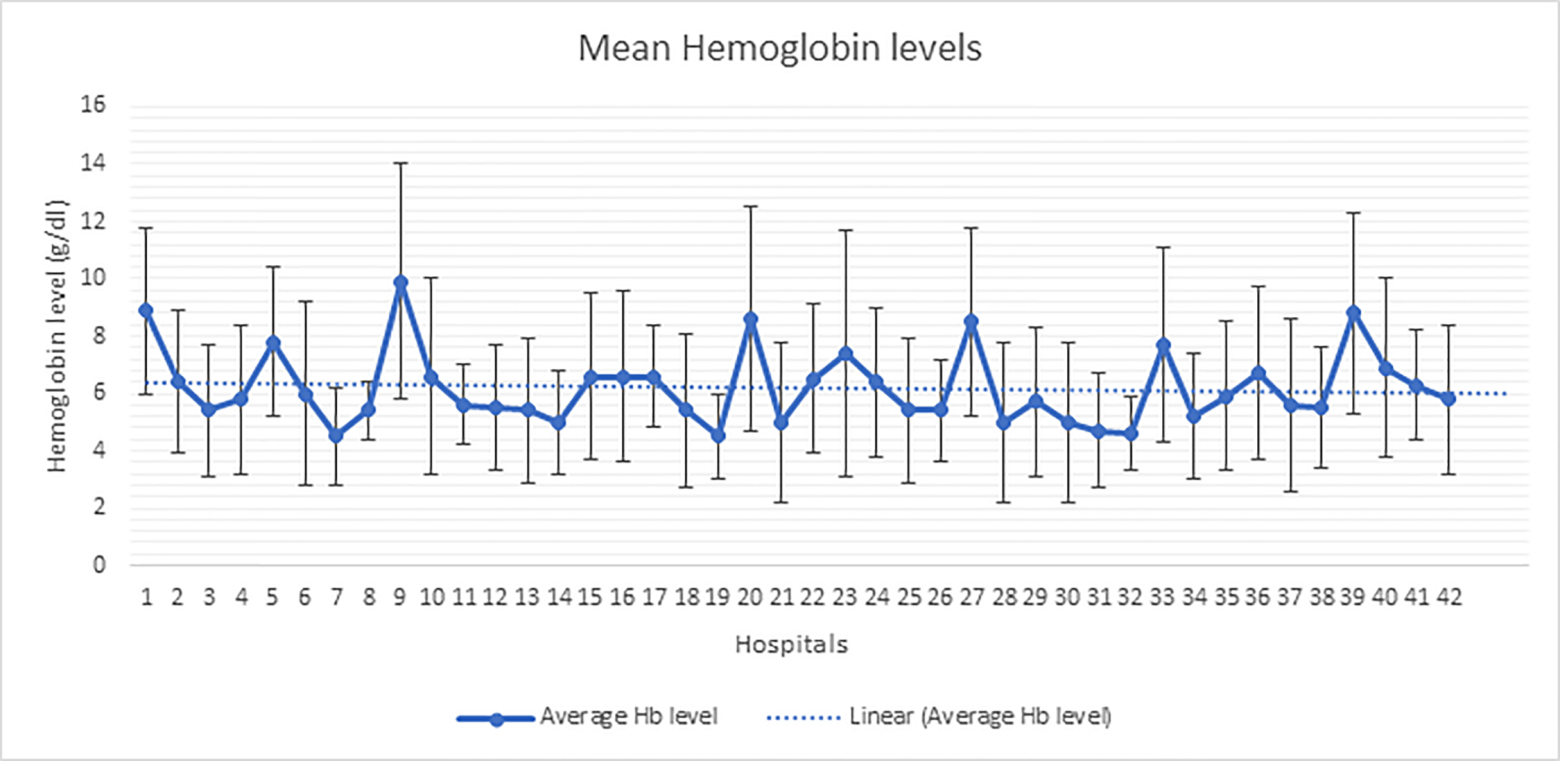
The average means of hemoglobin level across the hospitals (N = 42).

Fig 3, shows the variability of mean haemoglobin level across hospitals among blood requests with an exception of those from neonates. Majority of these blood requests are above the recommended WHO guidelines for minimum level of haemoglobin (4g/dl) for blood requests. The calculated average mean haemoglobin across the hospitals is 6.7 (sd±3.2) g/dl. The graph shows the average mean haemoglobin level for each hospital.

### Proportion of inappropriate blood requests

The overall proportion of inappropriate blood requests was 28.8% whereby all 42 hospitals had at least one inappropriate blood request. Within the hospital, an average of 28.9% had at least one inappropriate blood requests (Table **2**).

### Factors associated with inappropriate blood request

There was a significant association between inappropriate blood request with age group, pallor and type of ward patients admitted to (Table 3). Other factors significantly associated with inappropriate blood requests were; sex, pregnancy, admission due to injury, type of hospital ownership, underlying cause of anaemia, surgery on hospitalization, type of surgery, hospital area, malaria, respiratory distress, capillary refill, tachycardia, pallor, cold extremities and heart failure. (Table **3**).

**Table 3:** The variables associated with inappropriate blood request (N = 11,494).

Variables	Inappropriate (N = 11,494)	
	n (%)	P-value
**Sex (n = 12,171)**		<0.0001
Male	1,410 (31.9)	
Female	2,103 (27.1)	
**Age group (years) (n = 11,537)**		<0.0001
0–4	1,061 (60.8)	
5–14	148 (11.8)	
15–24	466 (24.3)	
25–34	627 (24.3)	
35–44	438 (25.6)	
45–54	201 (23.2)	
55–64	168 (27.2)	
65+	302 (36.1)	
**Pallor (n = 12,204)**		<0.0001
Yes	2,094 (19.7)	
No	1,423 (90.4)	
**Ward Type (n = 14,585**		<0.0001
Adult surgery	940 (45.1)	
Adult medical	239 (8.34)	
Paediatric medical	1,038 (35)	
Paediatric surgical	178 (51.9)	
ICU	91 (46.2)	
Obstetrics	747 (29.8)	
Gynaecology	254 (22.2)	
Orthopaedics	22 (47.8)	
Emergency department	1 (14.3)	
**Surgery on hospitalization (N = 12,204)**		0.002
Yes	697 (26.4)	
No	2,820 (29.5)	
**Type of ownership**		<0.0001
Government	2,529 (26.7)	
Private	988 (37.8)	
**Reported on capillary refill (12,166)**		<0.0001
No	3,043 (34)	
Yes	461 (14.4)	
**Reported on pallor (N = 12,131)**		<0.0001
No	1,423 (90.4)	
Yes	2,042 (19.3)	
**Level of haemoglobin**		<0.0001
Normal	522 (51.3)	
Anaemia	1,893 (29.7)	
**Reported tachycardia (N = 11,976)**		<0.0001
No	2,439 (38.1)	
Yes	1,004 (18)	

### Multivariable multilevel analysis

**Fig 4:**
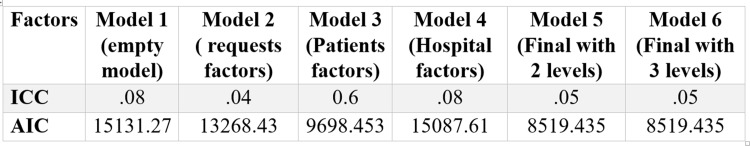
Model comparison.

A two-level mixed effects model was used to analyse the effects of patients’ individual characteristics and hospital-level factors in determining appropriateness of blood requests. From the empty model, 8% of the total variance in the risk of inappropriate blood request was accounted for by between-hospitals variation of characteristics (ICC = .08, p<0.0001). The between-hospitals variability declined over successive models, from 8% in the empty model to 6% in individual-level only model, but going back to 8% in hospital-level only model and 5% in the combined model. Thus, the combined model of individual-level (request and patient factor), and hospital-level factors was selected for predicting the risk of inappropriate blood request. ICC of 0.05, means 5% of variability of the risk of inappropriate blood request was explained by clusters (hospitals). Difference in hospital settings had little effect on inappropriate blood request (Fig **4**).

### Effect of individual factors

After adjusting for individual requests and hospital-level factors, reporting patient’s clinical signs was highly significantly associated with the risk of inappropriate requisition. Reporting pulse rate decreases the risk by 26% (RR 0.74; 95% CI 0.64, 0.84), and capillary refill by 27% (RR 0.73; 95% CI 0.63, 0.85) compared to not reporting them (Table **4**).

**Table 4:** Crude and adjusted regression of risk inappropriate blood requests among patients’ blood requests (N = 7201).

Characteristics	Crude		Adjusted	
	RR (95% CI)	P-value	RR (95%CI)	P-value
**LEVEL 1**				
**Tachycardia**				
Not-Reported	Baseline		Baseline	
Reported	0.40 (0.36, 0.45)	<0.0001	0.74 (0.64, 0.84)	<0.0001
**Capillary refill**				
Not-Reported	Baseline		Baseline	
Reported	0.51 (0.39, 0.49)	<0.0001	0.73 (0.63, 0.85)	<0.0001
**Surgery during hospitalization**				
No	Baseline		Baseline	
Yes	1.16 (1.02,1.3)	0.02	1.22 (1.06, 1.4)	0.006
**Pallor #Haemoglobin level^a^**				
**Effect of haemoglobin level**				
Not reporting pallor	Baseline		1.27 (1.03,1.58)	0.028
Reporting pallor	0.18 (0.17,0.2)		0.18 (0.17,2)	<0.0001
**Effect of reporting pallor**				
Normal haemoglobin level	Baseline		0.41 (0.32,0.51)	<0.0001
Anaemia	0.65 (0.58,0.72)		0.55 (0.49, 0.63)	<0.0001
**Age (years)**				
0–4	Baseline		Baseline	
5–14	0.15 (0.13, 0.18)	<0.0001	0.12 (0.1, 0.15)	<0.0001
15–24	0.33 (0.29, 0.37)	<0.0001	0.15 (0.1, 0.22)	<0.0001
25–34	0.32 (0.29, 0.35)	<0.0001	0.15 (0.1, 0.22)	<0.0001
35–44	0.34 (0.30, 0.38)	<0.0001	0.17 (0.11, 0.25)	<0.0001
45–54	0.29 (0.25, 0.34)	<0.0001	0.16 (0.1, 0.24)	<0.0001
55–64	0.34 (0.29, 0.4)	<0.0001	0.16 (0.1, 0.25)	<0.0001
65+	0.42 (0.37, 0.48)	<0.0001	0.19 (0.12, 0.28)	<0.0001
**LEVEL 2**				
**Ward type**				
General surgery	4.9 (4.2–5.7)	<0.0001	3.3 (2.7–4.2)	<0.0001
Adult medical	Baseline		Baseline	
Paediatric medical	4.5 (3.9–5.2)	<0.0001	1.5 (1.0–2.2)	<0.045
Paediatric surgical	5.5 (4.5–6.7)	<0.0001	1.8 (1.2–2.7)	<0.008
Intensive Care Unit	3.3 (2.6–4.3)	<0.0001	1.9 (1.3–2.7)	<0.001
Obstetrics	3.4 (3.0–4.0)	<0.0001	2.5 (2.0–3.1)	<0.0001
Gynaecology	2.7 (2.3–3.3)	<0.0001	2.1 (1.5–2.9)	<0.0001
Orthopaedics	4.3 (3.1–7.4)	<0.0001	3.8 (2.2–6.7)	<0.0001
Emergency department	1.3 (0.2–9.2)	0.8	1.8 (0.2–12.9)	0.566

Variables were adjusted for age, haemoglobin level, and surgery during hospitalization, reported tachycardia, capillary refill, pallor and ward type.

^a^ Interaction between reporting pallor and haemoglobin level

We examined inappropriate blood requisition related to surgical operations. Patients who had surgery during their hospital stay had 22% higher risk of inappropriate blood request comparing to those who had no surgery (RR 1.22; 95% CI 1.06, 1.4) (Table **4**).

We analysed the effect of confirming haemoglobin levels when the patient was pale on inappropriate blood requests. Reporting pallor of the patient, and a confirmed laboratory test of either low or normal haemoglobin levels decreases the risk by 82% (RR 0.18; 95%CI 0.17, 2.0) and 59% (RR 0.41; 95%CI 0.32, 0.51) respectively compared to not reporting pallor. (Table **4**).

We analysed the effect of age group on inappropriate blood requisition. Comparing to age group 0–4 years, patient aged betweeen5 and14 years had 88% lower risk of inappropriate requisition by o 88% (RR 0.12; 95% CI 0.1, 0.15) and those aged 65 years and above had 0.19 (RR 0.19; 95% CI 0.12, 0.28) times lower risk of inappropriate blood requisition, the rest were as shown in the table (Table **4**).

### Effect of hospital-level characteristics

The study aimed to show if the characteristics of the clusters (hospitals) in which patients were admitted would have an effect on inappropriate blood request, regardless of patients’ individual characteristics. After holding constant for the contribution of all the individual request level attributes, there was a significant association between the ward the patient was admitted to and inappropriate blood requisition in those hospitals. Being in surgical wards increased the risk by 3.3 times (RR 3.3; 95% CI 2.7, 4.2), paediatric medical by 1.5 times (RR 1.5; 95% CI 1.0, 2.2), paediatric surgical by almost 2 times (RR 0 1.8; 95% CI 1.2, 2.7) and obstetric ward by 2.5 times (RR 2.5; 95% CI 2.0, 3.1) higher risk of inappropriate blood request compared to being in medical ward. The rest as shown in the table (Table **4**).

## Discussion

The study involved 11,189 patients from whom 14,460 blood requests were placed in 42 different sampled transfusing facilities (hospitals). Out of those requests, 12,204 were conclusive in determining the inappropriateness of blood request. Of these, 3517 blood requests were inappropriate; a proportion of 28.8%. Similar findings were reported by Cheng and colleagues whereby they reviewed packed red blood cells (PRBC) cross-match requests and found inappropriate ordering was more pronounced for elective than emergency requests by 27.4% [30]. Several studies reported inappropriate requests among other blood components ranging from 22.2 to 42% [31–33]. All these studies show a large burden of inappropriate blood requests leading to unnecessary transfusions.

In this study factors which significantly predicted inappropriate blood requests were; age, not reporting clinical signs of the patient (tachycardia, capillary refill and pallor),confirmation of haemoglobin level, having surgery during the hospitalization period and ward type.

Findings show the blood requests for children below 5 years were at higher risk of being inappropriate comparing to other age groups. This was contrary to a systematic review study which reported inappropriate requests were higher at the older age of 65 years and above compared to ages below that [34]. In Tanzania as other tropical countries, malaria infections among under-fives is highly associated with anaemia [35,36], possibly contributing towards the increase demand for blood transfusion and hence higher chances of inappropriate blood requisitions [37,38].

The most important clinical signs to document are features of heart failure; decrease in capillary refills and increase in pulse rate. If these signs are well examined and documented, it would decrease the risk of inappropriate request and eventually inappropriate transfusion. These clinical signs have been the main indicators of transfusion in clinical settings [39,40], especially among children below five years [6,41].

This study has revealed that blood requests in which pallor is documented have decreased risk of inappropriate blood requests. Furthermore, when pallor is confirmed with haemoglobin level the risk of inappropriate request decreased significantly rather than when haemoglobin level only is used as an indicator for blood request. This is supported by several studies whereby it was found that a majority of inappropriate transfusions occurred among patients in which haemoglobin level was the only indicator for transfusion [42–44]. Since blood transfusions rely solely on the condition of the patient which is based on clinicians’ findings, we would then expect clearer documentation on these findings for those issuing the blood to make the best decision [45].

Having surgery during hospitalization had an effect on inappropriate blood request. Patients who had surgery had a sixteen times higher risk of their request being inappropriate than those who had no operation. Perioperative surgeries have been associated with over ordering of blood, consequently leading to wastage of blood [16,46,47]. Overall, surgical patients are more likely to be inappropriately transfused comparing to medical patients which has been associated with high mortality and morbidity [42,48]. This could be contributed by a perception that all surgical cases would need blood in some way. Furthermore bloodless surgery requires availability of proper infrastructures which are limited in developing countries [49].

This study further demonstrated being in surgical wards increased the risk of inappropriate blood requisition compared to being in medical wards and furthermore being in paediatric surgical wards. Similar findings have been reported on associations between inappropriate transfusion and elective and emergency transfusions in obstetrics, gynaecology and urology departments compared with other departments [30,42]. However in a review by International Consensus Conference on Transfusion Outcomes (ICCTO), there was no difference in inappropriate transfusion among hospital setting (ward type) [34]. Furthermore, we could argue on the basis of the knowledge of the clinician [50] of which this could not be ascertained in this study.

### Strengths and limitations

This study had a large sample size and is the first to explore the relationship between factors influencing inappropriate blood requests in Tanzania using country sample data. Although this study used all available information from the parent study, some of observations had missing values, which were found to be completely at random. There is a confidence in the generalizability of the results as hospitals were randomly selected. The parent study used design-standardized questionnaires consisting of closed-ended, easy-to-understand questions with appropriate response options. This decreases the likelihood that the interviewer would “interpret” the questions for the subject or will need to “probe” the subject for an appropriate response. Hence, from that it was assumed that observer bias was not a concern.

The study also had some limitations. Firstly, we found some outlier values such as age and haemoglobin level, which in many settings can be verified against the patients’ files. However, since we used a secondary dataset, this was not possible. Secondly, the use of secondary data did not allow us to analyse the effects of the various factors that might have influenced inappropriate blood request, such as clinician’s knowledge about blood transfusion and the experience on transfusion practices. Lastly, criteria for inappropriate blood requests were a proxy measure from inappropriate blood transfusion.

### Conclusions and recommendations

The proportion of inappropriate blood requisition is high which means a majority of transfusions that took place during the study period were unnecessary. The most significant risk factors of inappropriate requisition were; not reporting clinical presentation of the patients (tachycardia, pallor and peripheral capillary refills), and haemoglobin level, age of the patient and type of ward of admission. The combination of both laboratory findings (haemoglobin level) and physical findings (pallor) are necessary for making decisions on appropriate requests.

Due to the high prevalence of inappropriate blood requests there is a need to emphasize hospital transfusion committees to review their local transfusion guidelines and encourage the clinicians to abide to the national guidelines. This may help in making thorough physical and laboratory investigations prior to requesting blood for transfusions.

It is critical that hospital blood banks abide to transfusion guidelines by scrutinizing the blood requests to monitor blood distribution and prioritize requests for those in real need.

Future studies should be done to understand the effects of various factors that might influence inappropriate blood request, such as clinician’s knowledge about blood transfusion and experiences of transfusion practices.
